# Autism-Like Behaviours and Memory Deficits Result from a Western Diet in Mice

**DOI:** 10.1155/2017/9498247

**Published:** 2017-06-08

**Authors:** Ekaterina Veniaminova, Raymond Cespuglio, Chi Wai Cheung, Alexei Umriukhin, Nataliia Markova, Elena Shevtsova, Klaus-Peter Lesch, Daniel C. Anthony, Tatyana Strekalova

**Affiliations:** ^1^Department of Neuroscience, School for Mental Health and Neuroscience, Maastricht University, Universiteitssingel 40, 6200 MD Maastricht, Netherlands; ^2^Institute of Molecular Medicine, Sechenov First Moscow State Medical University, Moscow 119991, Russia; ^3^Faculty of Medicine, Neuroscience Research Center of Lyon, C. Bernard University, 8 Av. Rockefeller, 69373 Lyon, France; ^4^Department of Anaesthesiology, Queen Mary Hospital, The University of Hong Kong, 102 Pokfulam, Hong Kong; ^5^Department of Normal Physiology, Sechenov First Moscow State Medical University, Moscow 119991, Russia; ^6^Laboratory of Biomolecular Screening, Institute of Physiologically Active Compounds, Russian Academy of Sciences, Moscow Region, Russia; ^7^Division of Molecular Psychiatry, Laboratory of Translational Neuroscience, Department of Psychiatry, Psychosomatics and Psychotherapy, University of Würzburg, Fuechsleinstrasse 15, 97080 Würzburg, Germany; ^8^Department of Pharmacology, Oxford University, Mansfield Road, Oxford OX1 3QT, UK

## Abstract

Nonalcoholic fatty liver disease, induced by a Western diet (WD), evokes central and peripheral inflammation that is accompanied by altered emotionality. These changes can be associated with abnormalities in social behaviour, hippocampus-dependent cognitive functions, and metabolism. Female C57BL/6J mice were fed with a regular chow or with a WD containing 0.2% of cholesterol and 21% of saturated fat for three weeks. WD-treated mice exhibited increased social avoidance, crawl-over and digging behaviours, decreased body-body contacts, and hyperlocomotion. The WD-fed group also displayed deficits in hippocampal-dependent performance such as contextual memory in a fear conditioning and pellet displacement paradigms. A reduction in glucose tolerance and elevated levels of serum cholesterol and leptin were also associated with the WD. The peroxisome proliferator-activated receptor gamma coactivator 1-alpha (PPARGC1a) mRNA, a marker of mitochondrial activity, was decreased in the prefrontal cortex, hippocampus, hypothalamus, and dorsal raphe, suggesting suppressed brain mitochondrial functions, but not in the liver. This is the first report to show that a WD can profoundly suppress social interactions and induce dominant-like behaviours in naïve adult mice. The spectrum of behaviours that were found to be induced are reminiscent of symptoms associated with autism, and, if paralleled in humans, suggest that a WD might exacerbate autism spectrum disorder.

## 1. Introduction

In the context of increasing societal preference for “cafeteria-type diets” and “comfortable food” enriched with unsaturated fat and sugars, as well as the so-called “Western-type diet”, which is predominantly based on a heightened intake of unsaturated fat and cholesterol, there is an urgent need to study the physiological consequences of these diets. Excessive consumption of the Western diet has been shown to generate obesity, insulin resistance, hypercholesterolemia, and neuroinflammation in many organs, including the brain [1–6]. In addition, a Western-type diet may impact on reciprocal cognition [5, 7] and social interactions [3]. Human and animal studies suggest important roles of increased fat-/cholesterol-containing dietary regimen in behavioural abnormalities associated with social behaviour, aggression, and brain plasticity [7–12].

Recent experiments showed aberrant social interactions and increased measures of aggression associated with diets containing high amounts of fat and cholesterol. Dietary exposure to increased amounts of cholesterol and fat was shown to increase male aggression in monkeys [13], elevate the risk of autism spectrum disorder in offspring of humans approximately 1.5 times [14], and suppress social exploratory interactions in offspring of mice [15, 16]. Also, a combination of elevated contents of cholesterol and unsaturated fat was found to aggravate cognitive rigidity and social deficiency in the mouse model of autism [3]. Yet, these and other recently reported results provide limited information regarding the effects of a “Western”/westernized diet on social behaviours of adult individuals not predisposed to any abnormalities, including the changes typical for autism-like spectrum disorder.

The mechanisms that underlie deficient social interactions and brain plasticity associated with westernized diets can overlap. Increased rates of aggression-like traits are suggested to be related to reduced cognitive control and deficiencies in cognitive functions in general [17, 18]. Most of available literature reports decreased learning abilities in animals exposed to high-fat and high-cholesterol diets. Housing of rats on a high-cholesterol and fat-containing diet containing 2% cholesterol for 4 months led to impaired learning in the Morris water maze [19]; a similar diet induced altered stress response [20]. Spatial learning in an 8-arm maze was shown to be impaired in mice and rats housed on high-cholesterol and fat-containing diets [9, 12, 21, 22]. A high-fat diet containing 2% cholesterol induced deficits in contextual conditioning that were accompanied by altered hippocampal structure, such as a reduction in microtubule-associated proteins and elevation of markers of microglial activation in a rat [21]. Yet, very few studies addressing the effects of a westernized-type diet in the fear conditioning paradigms have been reported so far, while this paradigm provides higher sensitivity than other models to explore the integrity of hippocampal functions [23]. In addition, a high-fat/high-cholesterol diet was also associated with increased immobility in the forced swim test indicating that the diet also generates a negative affect [21].

Several metabolic endpoints in the high-cholesterol diet-fed mice have been noted that may underpin the behavioural changes, and these include increased insulin resistance, which is associated with a reduction in the expression of peroxisome proliferator-activated receptor gamma coactivator 1a (PPARGC1a), a marker of mitochondrial disbalance and impaired mitochondrial activity in dietary-induced type 2 diabetes [16, 24–26]. Diminished expression of this gene has been reported in the human hippocampus in patients with Alzheimer's disorder, and its expression level correlates with the clinical progression of dementia [27]. Mice genetically lacking the PPARGC1a gene exhibit an imbalance between inhibitory and excitatory synaptic transmission in the hippocampus, a mechanism that is suggested to be an important pathogenetic factor of autism [28, 29]. Thus, cognitive and other behavioural deficits reported in this work on mice fed with a cholesterol-enriched diet can result not only from lowered mitochondrial activity that is associated with decreased PPARGC1a brain levels but also from the specific role of this molecule in synaptic plasticity.

Our previous studies in mice employing a high-fat/high-cholesterol diet to induce nonalcoholic fatty liver disease (NAFLD) revealed that the diet increased levels of impulsivity, behavioural despair, anxiety, and reduced exploration of novel objects [30–32]. However, to date, the effects of excessive dietary cholesterol on social behaviours and aggression were not studied in this model. Here, we exposed female C57BL/6J mice to the Western diet (WD) [30, 31] to investigate the impact of the diet on social interactions, hippocampal function, and metabolism in normal mice exposed to the westernized diet during their adulthood. We are able to report, for the first time, that experimental exposure of mice to the WD profoundly affects their social interactions, substantially suppressing social exploratory contacts, inducing dominant-like behaviours and aberrant patterns of social behaviour. This diet also caused a deficient hippocampus-dependent performance, increased locomotion, downregulated mitochondrial activity marker PPARGC1a in the brain but not in the liver, and induced glucose intolerance and hyperleptin/hypercholesterolemia in mice. Thus, under conditions of excessive intake of cholesterol which do not alter gross physiological measures such as body weight, substantial changes in behaviour, some of which are reminiscent of symptoms associated with autism, can occur.

## 2. Methods

### 2.1. Animals

Studies were performed using 3-month-old female C57BL/6J from Janvier, Charles River, France. Mice were housed five per cage during the study, under a 12 h light-dark cycle (lights on: 19:00 h) with food and water ad libitum and under controllable laboratory conditions (22 ± 1°C, 55% humidity). All experiments were carried out in accordance with the European Communities Council Directive for the care and use of laboratory animals (2010/63/EU) upon approval by the Ethical Committee of the C. Bernard University on animal care and welfare.

### 2.2. Study Design and Dietary Challenge

**Figure 1:**
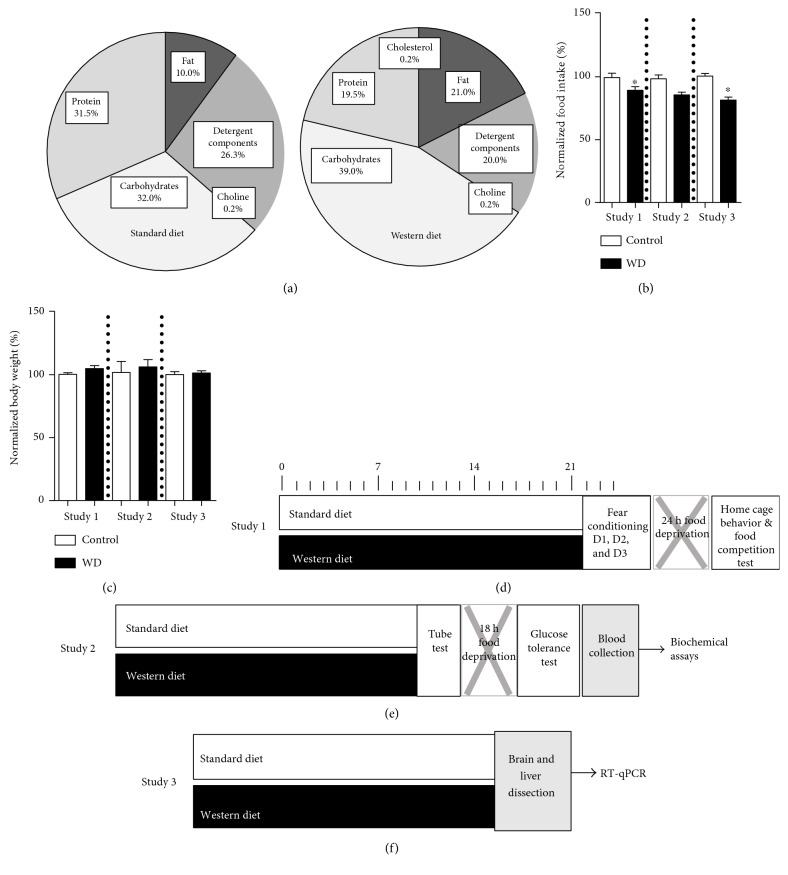
Experimental design. (a) Composition of the diets and percentage of total calories. Food intake (b) and body weight (c) normalized to control on day 21 of a dietary challenge (control: control diet, WD: high-cholesterol diet; ^∗^*p* < 0.05 versus that in the control diet, *t*-test). (d) Study of the effects of cholesterol-enriched diet exposure on mouse behaviour in fear conditioning test, home cage, and food competition test. (e) Study of the effects of cholesterol-enriched diet exposure on mouse performance in pellet displacement tube test, glucose tolerance, and blood biochemical parameters. (f) Study of the effects of cholesterol-enriched diet exposure on gene expression in the brain and liver.

Mice were fed with a regular laboratory diet with an energy content of 3.0 kcal/g, 6.55% unsaturated fat, and 32% carbohydrates (Mucedola s.r.l., Settimo, Italy) or with a diet containing 0.2% (*w*/*w*) cholesterol, 20% of saturated fat, 39% carbohydrates, and an energy content of 4.6 kcal/g, “Western diet” (WD), (Research Diet Inc., New Brunswick, NJ, USA) for three weeks as described elsewhere [30–32]. The content of the nutrients in calories and weight is indicated in Figure 1(a) and Supplementary Table 1 available online at https://doi.org/10.1155/2017/9498247. Mice from the two groups were compared for the parameters of social interaction, hippocampus-dependent performance, and several metabolic read-outs, in three separate experiments. Body weight and food intake were monitored weekly in each study, as described elsewhere (Figures 1(b) and 1(c); [30–32]) and were calculated as normalized to the mean of a control group.

After a three-week period of dietary challenge, a cohort of mice was studied for social interactions in a home cage and in a food competition test, as well as for an acquisition and an extinction of contextual fear in the fear conditioning paradigm (study 1; *n* = 14 for the control group and *n* = 20 for the group fed with a high-cholesterol and fat-containing diet; Figure 1(d)). Another cohort of animals was tested in the pellet displacement tube test, a rodent paradigm for a hippocampus-dependent performance [33, 34], and a glucose tolerance test, followed by blood collection for the analysis of leptin, triglyceride, and cholesterol content (study 2; *n* = 8 for the control group and *n* = 7 for the group fed with a high-cholesterol and fat-containing diet; Figure 1(e)). Finally, a portion of mice was sacrificed and dissected for the analysis of gene expression of a mitochondrial activity marker PPARGC1a, in the prefrontal cortex, hippocampus, hypothalamus, dorsal raphe, and liver (study 3; *n* = 5 for each group; Figure 1(f)). In the first two experiments, behavioural and biochemical assays were carried out during four consequent days following a period of a dietary intervention; gene expression was analysed at the midpoint of this testing period (Figures 1(d), 1(e), and 1(f)).

### 2.3. Behavioural Testing

All behavioural tests were carried out during an active period of animals' light cycle (09:00–21:00); behaviour was recorded and manually scored offline using behavioural criteria that were previously validated with automated scoring [35, 36]. The experimenter was blind for the diet used in the mice subjected to the testing.

#### 2.3.1. Social Interactions and Solitary Behavioural Activities in a Home Cage

In both experimental groups, social behaviours and other activities in a home cage were assessed 24 h after a food deprivation, immediately prior to the food competition test. Mice were food-deprived in order to potentiate their social interactions [32]. The top of a home cage was replaced by a transparent cover, and mouse behaviours were recorded during 10 min under the subtle lighting (light intensity 5 lux). The following behavioural parameters were evaluated: group huddle, “sitting alone”, time spent in motion, and digging behaviour. The group huddle was defined as sitting of a mouse in a physical contact to a body of at least one cage mate [36, 37]. “Sitting alone” defined as a position of a mouse in a cage without any physical contacts to another cage mate(s). Time spent in motion was defined as an appearance of horizontal movement of an animal where a position of a centre of a body changed in a cage with a speed > 2 cm/sec or an animal would display a rearing by taking a vertical position for >1 sec, as defined previously [38]. To evaluate digging (burrowing) behaviour, a species-specific behaviour of a displacement of bedding material using the snout and forepaws, percentage of mice that display this form of behaviour was calculated in each group; its total duration for an observation period was evaluated as well.

#### 2.3.2. Social Interactions during a Food Competition Test

In the food competition test, two 24 h food-deprived mice from different cages and the same experimental group were placed in a plastic observation cage (21 cm × 27 cm × 14 cm) which contained a piece of beef meat (2 g) for 10 min under subtle lighting (light intensity: 5 lux) and allowed to compete for food as described previously [32]. Our previous studies showed that these conditions, which were adapted from earlier studies [39], female mice which are genetically prone to display aggression, are triggered for agonistic behaviours, competition for food, and aggressive behaviours (Strekalova et al., *unpublished data*). While previous studies revealed a lack of direct evidences for proaggressive effects of a high-cholesterol diet on female mice in a food competition test [32], the same paradigm was used to study detailed analysis of social interactions in the current study.

During this test, animals were analysed for crawl-over behaviour, as well as body-body, nose-anal, and nose-nose contacts as described elsewhere [40]. The crawl-over behaviour was defined as climbing over the back and head of another animal. “Nose-nose” contacts were defined as maintaining vibrissae for longer than 1 sec. “Nose-anal” contacts were defined as an examination of the anogenital area of another mouse. “Body-body” contacts were defined as other physical contacts that did not fall under the criteria of above-described forms of interactions. For each type of social contacts, the latency, duration, and a number of behavioural events were scored.

Additionally, during a food competition test, each mouse was scored for horizontal activity by counting the number of lines crossed where squares were app 10 × 10 cm in size, for the entire observation period. Their vertical activity was scored by counting the total number of rearings; a latency to rear was registered as well.

#### 2.3.3. Acquisition and Extinction of Contextual Fear in the Fear Conditioning Paradigm

The apparatus (Open Science, Russia and Technosmart, Rome, Italy) consisted of a transparent plastic cubicle (25 × 25 × 50 cm) with a stainless-steel grid floor (33 rods, 2 mm in diameter). A single alternating electric current (AC, 50 Hz; 0.5 mA, 1 sec, Evolocus LLC, Tarrytown, NY, USA) was delivered after a 2 min acclimatization period. After delivery of the current, the mouse was immediately placed back in the home cage. Freezing behaviour was scored by visual observation during a test of memory recall that was carried out 24 h later as described elsewhere [41, 42]. The occurrence of freezing behaviour was assessed every 10 s for 180 s; each 10 s score was assigned to a freezing or nonfreezing period, and the percentage of time spent in freezing was calculated. Mice that spent ≥50% of time in freezing were defined as “good learners”. After scoring of memory recall, mice were left for another seven minutes in the apparatus for memory extinction; no foot shock was applied during this period. 24 h later, freezing behaviour was scored again in a 180 s recall of extinction session as in the previous trial and percentage of time spent in freezing was calculated.

#### 2.3.4. Pellet Displacement Tube Test

In order to further assess hippocampal function, all experimental groups were tested for pellet displacement in a tube test [33, 34]. A tendency to displace small objects, for example, small stones or food pellets from a tube inside the cage, is species-specific in mice and has been demonstrated to depend on an intact hippocampal formation [33]. Using a paper tube (internal diameter 4 cm, length 10 cm), filled with 20 food pellets and placed in the middle of a cage (21 cm × 27 cm × 14 cm), the latency to displace the first food pellet and time required for 50% and 100% tube emptying were assessed in mice.

### 2.4. Glucose Tolerance Test

The animals from both groups underwent an oral glucose tolerance test. Mice were fasted overnight during 12 h, starting at 20:00; thereafter, glucose solution (2 g/kg, 1.8 g/l) was delivered into the stomach by oral gavage and blood was sampled from the tail vein. Samples were obtained before glucose administration at time point 0 and 5, 15, 30, 60, 90, and 120 minutes after. The level of blood glucose was analysed using the OneTouch UltraEasy glucometer and strips (LifeScan OneTouch, Dubai, UAE). Because there were no group differences between basal blood glucose levels, absolute values of glucose concentrations were analysed. The area under a curve for the dynamics of this parameter was calculated as well.

### 2.5. Brain Dissection, RNA Extraction, and RT-qPCR

Mice were sacrificed by cervical dislocation as described elsewhere [30, 40]. The brain of each mouse was dissected, and the prefrontal cortex was isolated and stored at −80°C until use. mRNA was extracted by using TRI Reagent (MRC, USA). First-strand cDNA synthesis was performed using random primers and Superscript III transcriptase (Invitrogen, Darmstadt, Germany); 1 *μ*g total RNA was converted into cDNA. Quantitative PCR for the peroxisome proliferator-activated receptor gamma coactivator 1-alpha (PPARGC1a) gene and the housekeeping gene glyceraldehyde 3-phosphate dehydrogenase (GAPDH) was performed using the SYBR Green master mix (Bio-Rad Laboratories, Philadelphia, USA) and the CFX96 Real-time System (Bio-Rad Laboratories, Philadelphia, USA). Sequences of primers used are indicated in Supplementary Table 2. Data were normalized to GAPDH mRNA expression and calculated as relative fold changes compared to that in control mice as described elsewhere [30, 40]. Results of qRT-PCR measurement were expressed as Ct values, where Ct is defined as the threshold cycle of PCR at which amplified product was 0.05% of normalized maximal signal. We used the comparative Ct method and computed the difference between the expression of the gene of interest and GAPDH in each cDNA sample (2^−ΔΔCt^ method). Data are given as expression folds compared to the mean expression values in control mice.

### 2.6. Blood Biochemical Analysis

Trunk blood was collected by decapitation during animals' sacrifice, stored at 4°C overnight, and centrifuged at 10000*g* for 10 min at 4°C. Serum was collected and stored at −20°C until use. A commercially available Mouse Leptin (OB) ELISA Kit (Sigma-Aldrich, MA, USA) was used to measure leptin level; the optical densities of experimental plates were measured at 450 nm using a plate reader (Wallac 1420 VICTOR, Waltham, MA, USA). All samples were run in duplicate. Quantitative determination of cholesterol and triglycerides in mouse serum was performed on a biochemistry analyzer Konelab 30i (Thermo Fisher Scientific, MA, USA) using a cholesterol kit and triglyceride kit (Thermo Fisher Scientific). All procedures were done according to the instruction manual.

### 2.7. Statistics

Data were analysed using GraphPad Prism version 5.0 (San Diego, CA, USA). All quantitative data sets were first analysed for normal distribution using Shapiro-Wilk normality test; then, *t*-test and two-way ANOVA with Bonferroni post hoc testing were used for normally distributed data and Mann-Whitney test for not normally distributed data. Two-way ANOVA was used to analyse the results from the glucose tolerance test. Fisher's exact test was used for categorical data. The level of significance was set at *p* < 0.05.

## 3. Results

### 3.1. Changes in Food Intake and Body Weight

In accordance with the previous findings, intake of the high-cholesterol and fat-containing diet was lower than that of the control diet, likely due to the adjustment of mice to the higher caloric value of the WD. This difference was significant in studies 2 and 3 (*t* = 3.35, *p* = 0.004 and *t* = 5.97, *p* = 0.0001, respectively, Figure 1(a)) and close to a level of significance in study 2 (*t* = 2.18, *p* = 0.061). Body weight did not differ significantly between groups in all three studies, suggesting that the dietary intervention did not grossly alter body composition (*p* > 0.05, *t*-test, Figure 1(b)); however, a tendency to an increased body mass was found in study 1 (*t* = 1.79, *p* = 0.093). These findings are in line with our previous results that showed a lack of body weight changes and a decrease in intake of a high-cholesterol and fat-containing diet [24–26].

### 3.2. Aberrant Social Interactions, Hyperlocomotion, and Excessive Digging Behavior in Home Cage Conditions and during the Food Competition Test in Dietary-Induced Mice

**Figure 2:**
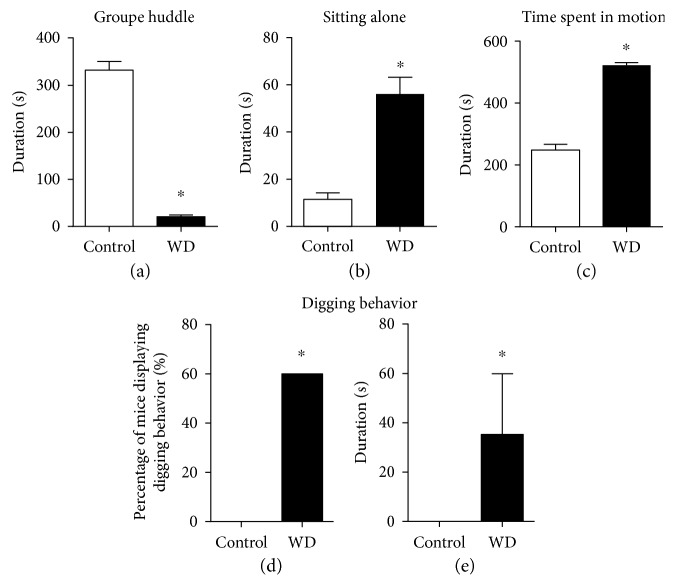
Dietary challenge with cholesterol results in aberrant home cage social behaviour. In comparison to control mice, the dietary-challenged group displayed (a) a significant decrease in the duration of group huddle, (b) a significant prolongation of the duration of “sitting alone” behaviour, and (c) a significant increase in the time spent in motion in the cage (^∗^*p* < 0.05 versus that in the control group, *t*-test). As compared to control animals, a group fed with the cholesterol-enriched diet showed a significant elevation of (d) the percentage of mice displaying digging behaviour (^∗^*p* < 0.05 versus that in the control group, Fisher's exact test) and (e) the duration of digging behaviour (^∗^*p* < 0.05 versus that in the control group, Mann-Whitney test). Control—standard diet, WD—high-cholesterol diet. Data are shown as mean ± SEMs.

During study 1, we found that in a home cage, in comparison with controls, mice fed with the high-cholesterol and fat-containing diet showed a significant decrease in the duration of group huddle behaviour (*t* = 16.49, *p* < 0.001, *t*-test; Figure 2(a)) and a significant increase in the duration of “sitting alone” behaviour (*t* = 5.81, *p* < 0.001, *t*-test; Figure 2(b)), as well as the time spent in motion (*t* = 13.39, *p* < 0.00, *t*-test; Figure 2(c)). The number of animals expressing digging behaviour during this test and duration of digging (burrowing) behaviour, which is a sign of active copying and a correlate of social dominance, were significantly increased in the group fed with the fat-/cholesterol-enriched diet compared to the control (*p* = 0.003, Fisher's exact test and *U* = 24.00, *p* = 0.003, Mann-Whitney test, Figures 2(d) and 2(e)). Thus, consumption of the high-cholesterol and fat-containing diet induces social avoidance, hyperlocomotion, and excessive digging behavior in home cage conditions.

**Figure 3:**
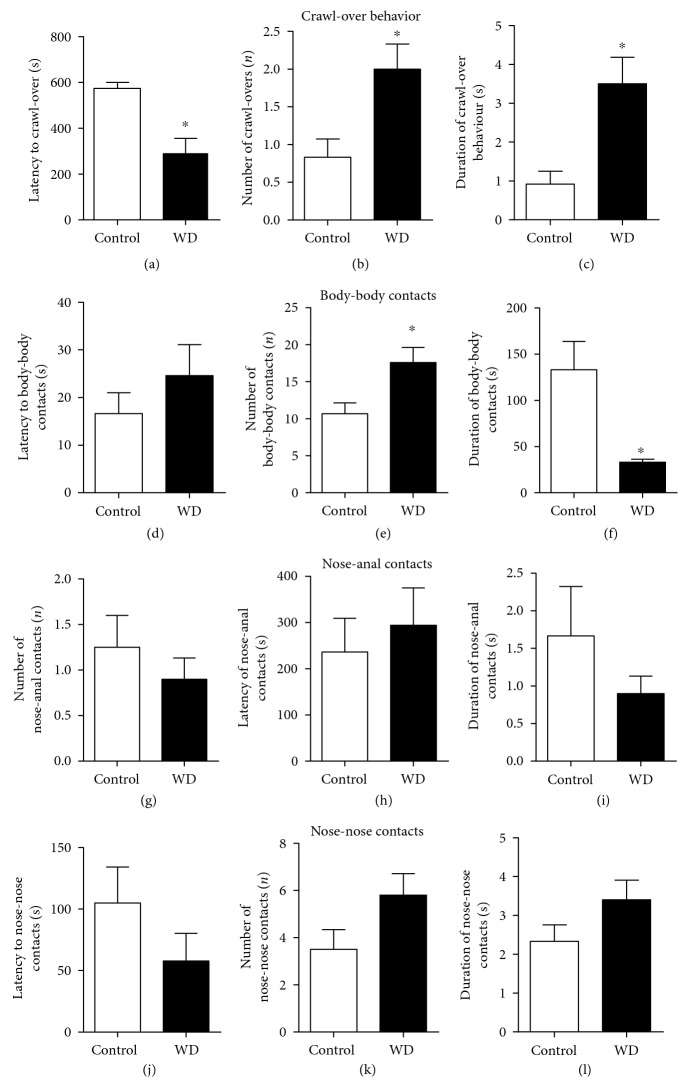
The high-cholesterol diet potentiates dominant-like behaviour and suppresses sociability in a food competition test. In comparison to the control group, mice housed on the high-cholesterol diet showed (a) a significant decrease in the latency to crawl-over behaviour and (b) a significant increase in a number of crawl-overs, as well as (c) prolonged total duration of crawl-over behaviour (^∗^*p* < 0.05 versus that in the control group; Mann-Whitney test). In comparison to the control group, mice exposed to the cholesterol-enriched diet had (d) no significant changes in the latency to body-body contacts and had (e) a significant increase in a number of body-body contacts (^∗^*p* < 0.05 versus that in the control group; Mann-Whitney test) and (f) a significant decrease in the duration of body-body contacts (^∗^*p* < 0.05 versus that in the control group; Mann-Whitney test). No significant group differences were observed between the groups in the parameters of nose-anal contacts: (g) the number of nose-anal contacts, (h) latency to nose-anal contacts, and (i) total duration of nose-anal contacts (^∗^*p* > 0.05 versus that in the control group; Mann-Whitney test). There were no significant group differences in the parameters of nose-nose contacts: (j) latency of nose-nose contacts, (k) number of nose-nose contacts, and (l) total duration of nose-nose contacts (^∗^*p* > 0.05 versus that in the control group; Mann-Whitney test). Control—standard diet, WD—high-cholesterol diet. Data are shown as mean ± SEMs.

During the food competition test, in comparison with controls, the dietary-challenged group showed a significant decrease in the latency of crawl-over behaviour (*U* = 25.50, *p* = 0.020; Mann-Whitney test, Figure 3(a)) and a significant increase in the number of crawl-over behavioural events (*U* = 25.50, *p* = 0.020, Mann-Whitney test, Figure 3(b)) and the duration of this behaviour (*U* = 19.00, *p* = 0.005, Mann-Whitney test, Figure 3(c)). In comparison to mice housed on a regular diet, mice fed with the high-cholesterol and fat-containing diet showed no difference in the latency of body-body contacts between the groups (*U* = 10.00, *p* = 0.398; Mann-Whitney test, Figure 3(d)); however, the number of the body-body contacts was significantly increased (*U* = 3.00, *p* = 0.026; Mann-Whitney test, Figure 3(e)), while the total duration of this behaviour was decreased (*U* = 1.00, *p* = 0.009, Mann-Whitney test, Figure 3(f)). The latency, number, and duration of nose-anal and nose-nose contacts were not different between the groups (*U* = 49.50, *p* = 0.502; *U* = 53.50, *p* = 0.615; *U* = 53.50, *p* = 0.615 and *U* = 10.00, *p* = 0.416; *U* = 7.00, *p* = 0.171; *U* = 8.00, *p* = 0.197, respectively; Mann-Whitney test, Figures 3(g), 3(h), 3(i), 3(j), 3(k), and 3(l)).

**Figure 4:**
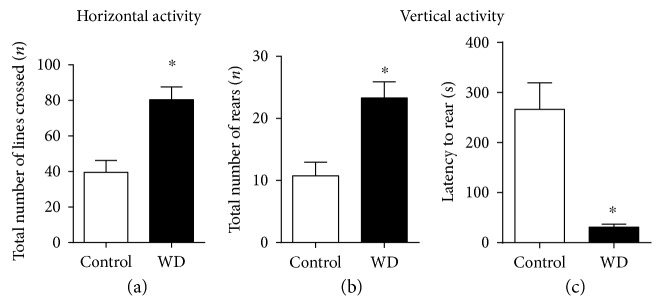
The cholesterol-enriched diet enhances horizontal and vertical activity during food competition test. In comparison to control mice, animals exposed to the cholesterol-enriched diet showed (a) a significant increase in the total number of line crossing (^∗^*p* < 0.05 versus that in the control group, *t*-test), (b) significant elevation of the total number of rearings (^∗^*p* < 0.05 versus that in the control group, Mann-Whitney test), (c) and a significant decrease in the latency of rearings (^∗^*p* < 0.05 versus that in the control group, *t*-test). Control—standard diet, WD—high-cholesterol diet. Data are shown as mean ± SEM (a, c) and median with interquartile range (b).

During the food competition test, in comparison with control mice, the group fed with the high-cholesterol and fat-containing diet showed a significant increase in the total number of line crossings (*t* = 4.18, *p* < 0.001, *t*-test, Figure 4(a)) and in the total number of rearings (*U* = 17.50, *p* = 0.004, Mann-Whitney test, Figure 4(b)) and a significant decrease in the latency of the first rearing event (*t* = 4.42, *p* = 0.001, *t*-test, Figure 4(c)). Together, these data suggest increased dominant-like behaviours, reduced sociability associated with abnormal social behaviour, and hyperlocomotion in mice housed on the WD.

### 3.3. Exposure to a Diet Enriched with Fat and Cholesterol Results in the Deficient Hippocampus-Dependent Performance in the Fear Conditioning and Pellet Displacement Tests

**Figure 5:**
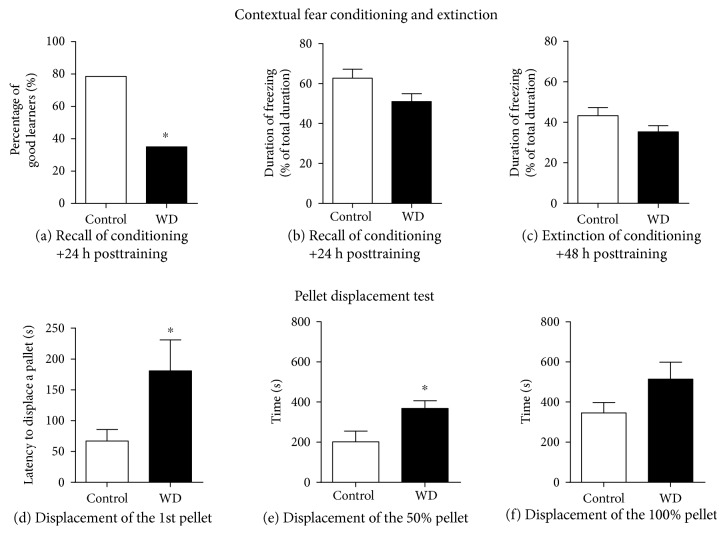
The high-cholesterol diet compromises the hippocampus-dependent performance. In the fear conditioning test, in comparison to control mice, the dietary-challenged group showed (a) significant decreased number of “good learners” (^∗^*p* < 0.05 versus that in the control group, Fisher's exact test), (b) a strong tendency to a reduced time spent with freezing during recall of conditioning (*p* = 0.055 versus that in the control group, *t*-test), and (c) a significant decrease in time spent with freezing in a memory extinction protocol (^∗^*p* < 0.05 versus that in the control group, *t*-test). In the pellet displacement tube test, in comparison to control animals, mice exposed to the cholesterol-enriched diet showed significantly prolonged (d) latency of a displacement of the 1st pellet and (e) the duration of displacement of 50% pellets (^∗^*p* < 0.05 versus that in the control group, *t*-test) and (f) did not differ in the duration of a displacement of 100% pellets (*p* > 0.05 versus that in the control group, *t*-test). Control—standard diet, WD—high-cholesterol diet. Data are shown as mean ± SEM.

In the fear conditioning test, in comparison to that in control mice, the number of “good learners” defined by the percentage of time spent with freezing ≥ 50% at the memory recall session was significantly diminished in the dietary-challenged group (*p* = 0.017, Fisher's exact test, Figure 5(a)); also, there was a strong trend to a decreased duration of freezing in this group (*t* = 1.96, *p* = 0.059, *t*-test, Figure 5(b)), suggesting reduced contextual memory in mice housed on the high-cholesterol and fat-containing diet. During the recall of memory extinction, mice housed on the WD showed significantly shorter duration of freezing than control mice, suggesting faster extinguishing of contextual memory due to its weaker acquisition (*t* = 3.024, *p* = 0.005, *t*-test, Figure 5(c)). During study 2, in the pellet displacement tube test, the latency to displace a pellet and time required for a 50% emptying the tube with pellets were significantly increased in the high-fat/high-cholesterol diet group, in comparison to control animals (*t* = 2.26, *p* = 0.044 and *t* = 2.62, *p* = 0.020, respectively, *t*-test, Figures 5(d) and 5(e)). The time required for a 100% emptying of the tube did not differ significantly between the groups (*t* = 1.74, *p* = 0.105, *t*-test, Figure 5(f)). Together, these data suggest a moderate deficiency in the hippocampus-dependent performance in mice fed with the high-cholesterol and fat-containing diet.

### 3.4. Altered Central and Peripheral Metabolic Markers in Mice Housed on the High-Cholesterol and Fat-Containing Diet

**Figure 6:**
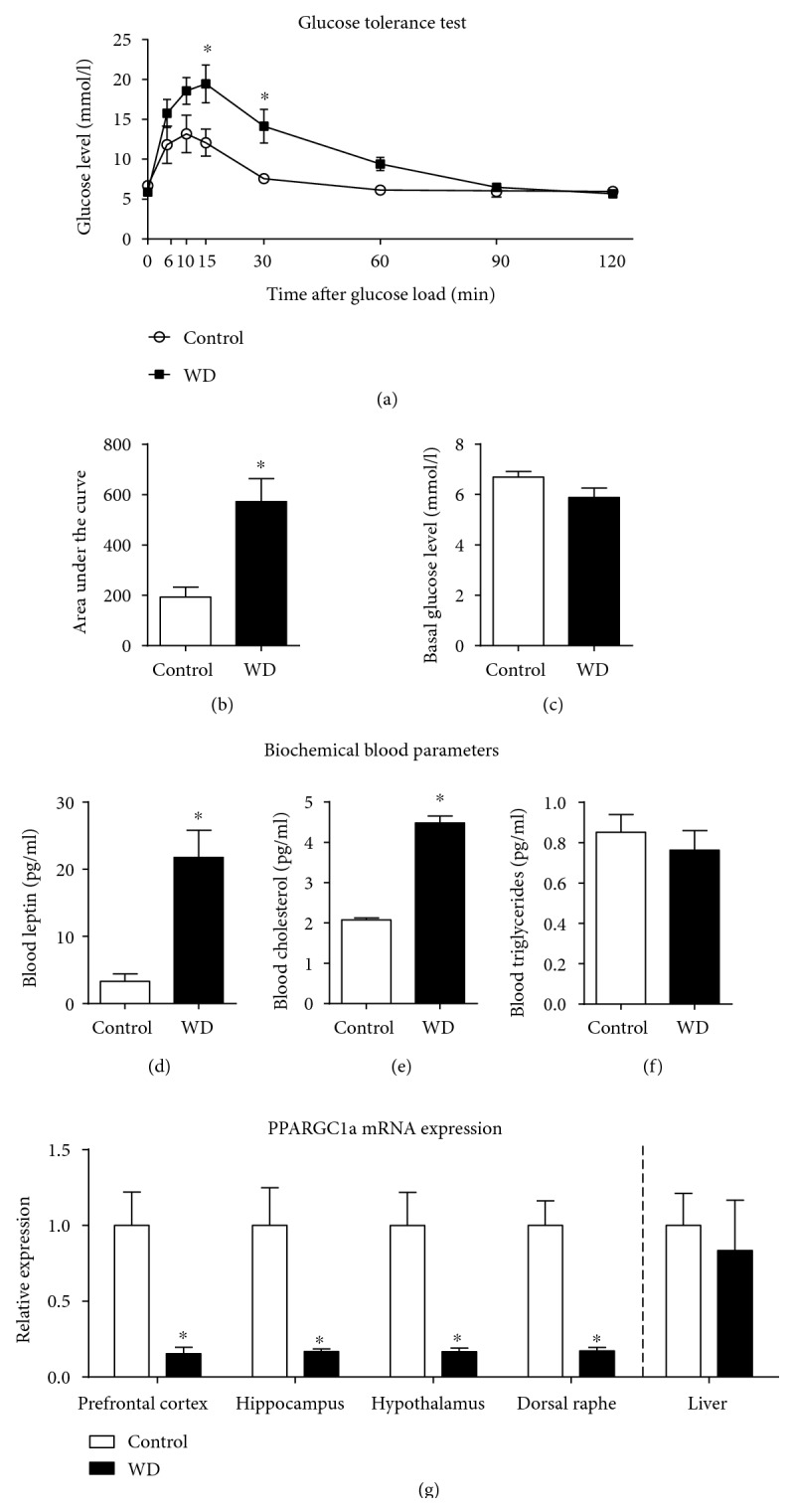
Effects of high-cholesterol diet exposure on glucose tolerance, biochemical blood parameters, and PPARGC1a gene expression. (a) In comparison to that in the control, there was an increase in blood glucose level at 15 and 30 min after glucose load in the glucose tolerance test (^∗^*p* < 0.05 versus that in the control group, 2-way ANOVA and Bonferroni post hoc test). (b) There was a significant increase in the area under curve in glucose concentration in dietary-challenged mice in comparison to controls (^∗^*p* < 0.05 versus control group, *t*-test). (c) There was no significant difference in basal glucose levels between mice housed on the standard and high-cholesterol diets. Mice housed on the high-cholesterol diet, as compared to the control group, showed significantly increased (d) blood leptin levels, (e) blood cholesterol levels (^∗^*p* < 0.05 versus that in the control group, *t*-test), and (f) unaltered blood level of triglycerides (*p* > 0.05 versus that in the control group, *t*-test). (f) In comparison to control mice, animals fed with the cholesterol-enriched diet had significantly decreased PPARGC1a mRNA in all brain areas (^∗^*p* < 0.05 versus that in the control group, 2-way ANOVA and Bonferroni post hoc test) but not in the liver (*p* > 0.05 versus that in the control group, 2-way ANOVA and Bonferroni post hoc test). Control—standard diet, WD—high-cholesterol diet. Data are shown as mean ± SEM.

At study 2, in the glucose tolerance test, two-way ANOVA revealed a significant effect of both the diet and time after glucose load on blood glucose level (*F* = 10.16, *p* = 0.013 and *F* = 19.15, *p* < 0.001; Figure 6(a)). In comparison with control mice, the dietary-challenged group showed a significant increase in blood glucose levels at the time points 15 and 30 min of the test and a tendency to increase at time point 10 min (10 min: *t* = 2.54, *p* = 0.108; 15 min: *t* = 3.48, *p* = 0.007; 30 min: *t* = 3.09, *p* = 0.024, Bonferroni test), suggesting a decrease in glucose tolerance in the latter group. No significant group differences were found at other time points. The area under a curve calculated for a 2 h period of the afterload measurements of blood glucose levels normalized to the baseline was significantly increased in the high-cholesterol and high-fat diet group as compared to the control (*t* = 3.21, *p* = 0.012, *t*-test, Figure 6(b)). There was no difference in basal glucose level between the groups (*t* = 1.67, *p* = 0.137, *t*-test; Figure 6(c)). Serum levels of leptin and cholesterol were significantly increased in the group fed with the high-cholesterol diet as compared to control animals (*t* = 4.40, *p* = 0.037 and *t* = 13.47, *p* < 0.001, respectively; *t*-test; Figures 6(d) and 6(e)); no difference was found in triglyceride level (*t* = 0.68, *p* = 0.529; *t*-test; Figure 6(f)).

Two-way ANOVA showed significant differences between the groups investigated in study 3 in the PPARGC1a mRNA concentrations in the brain (*F* = 56.12, *p* < 0.001), which were independent of the brain area (*F* = 0.28, *p* = 0.841; two-way ANOVA; Figure 6(g)). The PPARGC1a mRNA levels were significantly lower in all investigated brain regions in mice fed with the high-cholesterol and fat-containing diet, in comparison to control mice (prefrontal cortex: *t* = 4.00, *p* = 0.001; hippocampus: *t* = 3.92, *p* = 0.002; hypothalamus: *t* = 3.93; *p* = 0.002; and dorsal raphe: *t* = 3.13, *p* = 0.015, Bonferroni test). Hepatic PPARGC1a mRNA levels did not significantly differ between the groups (*t* = 0.42, *p* = 0.687, *t*-test).

## 4. Discussion

In this study, we showed, for the first time, that chronic exposure of naïve adult mice in the NAFLD model employed here to a high-cholesterol and fat-containing diet induces substantial changes in their social behaviour, comprising of a reduction in sociability and an increase in a dominant-like behaviour. These changes were accompanied by altered patterns of social behaviours and hyperactivity in a context of social interactions. Furthermore, dietary challenge with the high-cholesterol and fat-containing diet resulted in a decreased acquisition and enhanced extinction of contextual fear conditioning and slower hippocampus-dependent performance in pellet displacement tube test. Glucose intolerance, elevated plasma leptin and cholesterol levels, and overexpression of the PPARGC1a gene in the brain but not in the liver paralleled above-described behavioural abnormalities.

The analysis of social interactions both in a home cage and during a food competition test demonstrated that in comparison to control animals, dietary-challenged mice were less sociable, spending less time huddling and longer time “sitting alone” in a home cage, as well as shorter duration of body-body contacts during the food competition model. These data can be interpreted as a sign of social avoidance of both familiar and unfamiliar mice displayed by animals fed with the WD. They also rule out a factor of neophobia that could explain lower social interactions in cases where only the interactions with unfamiliar mouse would be reduced in mice exposed to the WD.

Our results are generally in line with several studies that reported diminished sociability in rodents after exposures to diets containing high amounts of fat/cholesterol; however, previous works largely dealt with their effects on the offspring [3, 15]. In one of the most recent studies, Buffington and colleagues found that the offspring of dams fed with a high-fat diet containing high amounts of cholesterol displayed markedly dropped number, frequency, and duration of social contacts both with familiar and unfamiliar mouse, as well as impaired long-term potentiation in the ventral tegmental area, a sign of a deficient synaptic plasticity [15]. These deficits were rescued by supplementary oxytocin and a restoration of normal microbiota parameters, which were affected in the offspring of dietary-challenged mice and can potentially mediate reported effects here. Moreover, another study with a high-fat diet containing high amounts of cholesterol showed that it can exacerbate social deficiency and cognitive rigidity in BTBR T+tf/J inbreed mouse line, a model of autism. BTBR mice, after housing on a high-fat diet containing high amounts of cholesterol starting at weaning, demonstrated greater deficits in social memory, lowered preference for social novelty, and impaired learning of the T-maze than these mice fed with a regular diet [3].

In the current study, the group fed with the WD revealed heightened scores of crawl-over behaviour in a food competition test and burrowing activity in a home cage, which are generally accounted for the manifestations of a dominant-like behaviour [40, 43]. Burrowing behaviour was shown to correlate with elevated levels of social dominancy and not be displayed by mice with subordinate social traits [43]. Of note, signs of burrowing behaviour were completely absent in the control group in the current study and were expressed only by dietary-challenged animals. Other studies suggest that burrowing behaviour is associated with repetitive behaviour and impulsivity that was recently reported in a study with C57 female mice fed with a fat and high-cholesterol diet [44].

In many studies with dietary interventions and assessment of social behaviours, a factor of a gain/loss of body weight was suggested to determine the outcome [45]. In the current work, we choose to apply a dietary challenge that is selectively enhanced in a content of cholesterol and also does not induce a change in body weight, as such, excluding a factor of obesity in the induction of negative consequences of dietary cholesterol. Thus, potential confounds related to altered body mass in the evaluation of social and other behaviours could be ruled out.

Our study has also revealed the alternations in the basic patterns of social interactions. An increase in the number of body-body contacts associated with a decrease in their duration can be interpreted as a sign of impulsive behaviour that was previously shown in an employed model under different experimental settings from those used in the present work, which did not include the elements of social interaction unlike the current work [30]. In line with this result and the previous findings, we found increased vertical and horizontal locomotion during both home cage testing and food competition test in cholesterol-challenged mice, suggesting their impulsivity which was displayed in a context of social interactions. Thus, mice challenged with high amounts of dietary cholesterol and fat display hyperlocomotion/impulsivity regardless of whether or not social element presents in their environment.

Similar to our results, a combination of exposure to a high-fat/high-cholesterol diet with food deprivation was recently found to enhance behavioural signs of impulsivity and upregulation of several molecular factors involved in the pathophysiology of impulsivity and addiction [45, 46]. Exposure of rodents to this type of diet has been shown to evoke substantial alternations in dopamine signalling in several brain structures including the prefrontal cortex [46, 47], the nucleus accumbens [47], and the hypothalamus [48]. As these changes have been associated with the mechanisms in social interactions, motivation, and various other behaviours [49–52], they can underlie the above-described behavioural signs of hyperactivity and impulsivity observed in our and previous studies.

Increased rates of dominant behaviours and decreased signs of sociability can be related to deficient cognitive functions in general and hippocampal plasticity in particular [17, 18]. The current study showed that applied exposure to a high-cholesterol diet reduces contextual fear conditioning in mice that is in line with the previous findings in this paradigm [21, 53]. Mice housed on a diet enriched with cholesterol were previously found to show increased anxiety [30, 31] generally known to potentiate fear learning. Yet, animals from this group displayed lowered percentage of “good learners” and a strong tendency to a reduction in freezing behaviour, suggesting deficient contextual learning. Significantly increased extinction of contextual conditioning as shown by diminished duration of freezing behaviour further suggests weaker learning abilities of mice housed on the high-cholesterol diet. Other animal studies found altered learning deficits in mice and rats that were housed on a high-cholesterol/high-fat diet and tested in a range of learning and memory tasks including water mazes, variable interval delayed alternation task, object recognition test, and operant bar-pressing task [7–12, 54, 55].

Findings described here in the fear conditioning test were further supported by the data obtained in the pellet displacement tube test. Dietary-challenged mice showed a slower performance of emptying a tube filled with pellets in this test, which is regarded as a sign of reduced hippocampus-dependent functions. Previous studies validated the food pellet displacement test as a paradigm, in which rodent behaviour of object displacement from a tube was found sensitive to the lesions of dorsal hippocampus [33] and accompanies the deficits in the hippocampal plasticity during depressive-like syndrome [34] and in a model of Alzheimer's disease [56]. Pellet displacement in rodents was shown to be suppressed by systemic inflammation [56, 57], a feature of animal models of a high-fat/high-cholesterol diet [6, 9, 26, 30].

As deficient cognition, reduced exploration, aggressive behaviour, attention deficit, depressive-like and anxiety-like changes, neophobia, and impulsivity constitute the elements of autistic behavioural profile [18, 58], behavioural changes in mice fed with the WD resembled proautistic behavioural repertoire.

Behavioural changes found in mice exposed to dietary cholesterol were accompanied by pronounced metabolic changes. We found increased blood concentrations of cholesterol and leptin, while the triglyceride level was unaltered. Dietary-induced hyperleptinemia and hypercholesterolemia were reported to be consistent in high-fat/high-cholesterol models [22, 59, 60], whereas similar to our study, the changes in blood triglyceride levels can vary [22, 59].

The present work has demonstrated delayed restoration of normal glucose levels after glucose load in the WD-challenged mice, suggesting their impaired glucose tolerance and insulin resistance that were not reported before in the NAFLD model employed here. Together with marketable inflammation that was previously demonstrated in an applied model here [30] and that can be a causal factor of insulin resistance [61], this finding led to suggesting altered insulin-mediated signalling in the brain, where insulin resistance in the hippocampus may account for reported behavioural abnormalities here. The use of high-fat/high-cholesterol diets showed that behavioural changes of rodents housed on these diets are accompanied by type II diabetes mellitus [8, 10, 21, 53]. Importantly, heightened occurrence of diabetes and reduced glucose tolerance during autistic spectrum disorder is well established in the literature [62, 63].

Epidemiological and clinical studies provide evidence for the role of metabolic syndrome on the occurrence of dementia and cognitive decline, in particular in the presence of inflammation [64, 65]. Mitochondrial dysfunction was found to be associated with social deficits in young individuals [62, 66, 67] and cognitive impairment in aging people and animals [68, 69]. Clinical and animal studies suggest a common neurobiological basis and interrelationship for aggression, diminished cognitive abilities, behavioural disinhibition, and metabolic abnormalities. Remarkably, pharmacological enhancement of insulin receptor signalling was demonstrated to rescue normal emotional behaviours in an employed paradigm here [31]. Since central and peripheral glucose intolerance is well documented to underlie numerous affective and cognitive abnormalities [70–74], this mechanism is likely to explain described behavioural aberrations here.

Our study revealed a decrease in PPARGC1a, a marker of diminished mitochondrial activity during dietary-induced type 2 diabetes [16, 31, 75, 76], in the brain of mice housed on the high-fat/high-cholesterol diet. This finding is in line with reported reduced glucose tolerance here in the WD group and further supports the view that suppressed mitochondrial functions in the brain may underlie described behavioural abnormalities here of mice housed on the WD. Interestingly, diminished expression of this gene was also found in the human hippocampus, correlating with clinical progression of dementia in patients with Alzheimer's disorder [27]. Mice genetically lacking the PPARGC1a gene exhibited an imbalance between inhibitory and excitatory synaptic transmission in the hippocampus, a mechanism that is suggested to be an important pathogenetic factor of autism [28, 77]. Thus, cognitive and other behavioural deficits reported in this work on mice fed with the cholesterol-enriched diet can result not only from lowered mitochondrial activity that is associated with decreased PPARGC1a brain levels but also specific roles of this molecule in synaptic plasticity.

Previously, we showed a decreased gene expression in an employed model here of another member of peroxisome proliferator-activated receptor family member, PPARGC1b, both in the brain and in the liver [31], whereas no changes in the hepatic PPARGC1a were found in the present study. These results highlight functional differences between two molecules as suggested earlier [31, 77].

Together, our data are generally consistent with so far obtained results with rodent models suggesting that diets containing high amounts of fat/cholesterol can evoke social deficiencies which resemble the features of autism spectrum disorders. Our results correspond to the current epidemiological findings, demonstrating a link between obesity and autism [14, 62], and to reports on ameliorated cognitive and behavioural symptoms of autism due to a diet [63]. Thus, the results reported here, along with currently accumulated data considering behavioural abnormalities in the light of proautistic changes, allow speculation that preference for the “Western diet” can be a potential risk factor for this spectrum of diseases in humans. These findings provide evidence for a possible environmental risk factor that can contribute to the production of autistic-like symptoms.

## Supplementary Material

Supplementary Data. Table 1: The composition of the diets with respect to the content of carbohydrates, saturated/unsaturated fat and protein, w/w. Table 2: Sequences of primers used.
