# Supplementary material for: Autism-Like Behaviours and Memory Deficits Result from a Western Diet in Mice
Source: Neural Plast. 2017 Jun 8;2017:9498247. doi: 10.1155/2017/9498247 (PMC5480052; doi:10.1155/2017/9498247)
Supplement: Supplementary file 1 — Supplementary Data. Table 1: The composition of the diets with respect to the content of carbohydrates, saturated/unsaturated fat and protein, w/w. Table 2: Sequences of primers used. [file 9498247.f1.docx]

**Supplementary Data**

**Table 1. The composition of the diets with respect to the content of carbohydrates, saturated / unsaturated fat and protein, w/w**

|  | **Standard chow** | **Western diet** |
| --- | --- | --- |
| Total fat | 10% | 21% |
| Saturated fat | 6.5% | 20% |
| Polyunsaturated and monounsaturated fat | 3.5% | 1 % |
| Carbohydrates: starch and sugars | 32.0% | 39.0% |
| Protein | 31.5% | 19.5% |
| Detergent components | App.26.3% | App.20.0% |
| Choline | 0.2% | 0.2% |
| Cholesterol (%) | 0% | 0.2% |

**Table 2. Sequences of primers used**

| **mRNA target** | Primer sequence (5' → 3') | |
| --- | --- | --- |
| **PPARGC1a** | Forward | CTCCAGTTCCGGCTCCTC |
|  | Reverse | CCCTGTGCTCTCACGTCTG |
| **Gapdh** | Forward | ACCCCTTCATTGACCTCAACTACATG |
|  | Reverse | CCTTCTCCATGGTGGTGAAGAC |
